# (Azido-κ*N*){(*E*)-2-[1-(pyridin-2-yl)ethyl­idene­amino]­phenolato-κ^3^ *N*,*N*′,*O*}copper(II)

**DOI:** 10.1107/S1600536813019570

**Published:** 2013-07-20

**Authors:** Amitabha Datta, Jack K. Clegg, Jui-Hsien Huang, Shiann-Cherng Sheu

**Affiliations:** a Department of Chemistry, National Changhua University of Education, Changhua 50058, Taiwan; b School of Chemistry and Molecular Biosciences, University of Queensland, Brisbane St Lucia, Queensland 4072, Australia; c Department of Occupational Health and Safety, Chang Jung Christian University, Tainan City 71101, Taiwan

## Abstract

In the title complex, [Cu(C_13_H_11_N_2_O)(N_3_)], the Cu^II^ cation is four-coordinated by an N_2_O donor set of the tridentate Schiff base ligand and by the terminal N atom of the azide anion, forming a slightly distorted square-planar configuration.

## Related literature

For related structures, see: Talukder *et al.* (2004 ▶); Sun (2008 ▶); Wang *et al.* (2012 ▶); Yu (2012 ▶). For the synthesis, see: Shita *et al.* (2009 ▶).
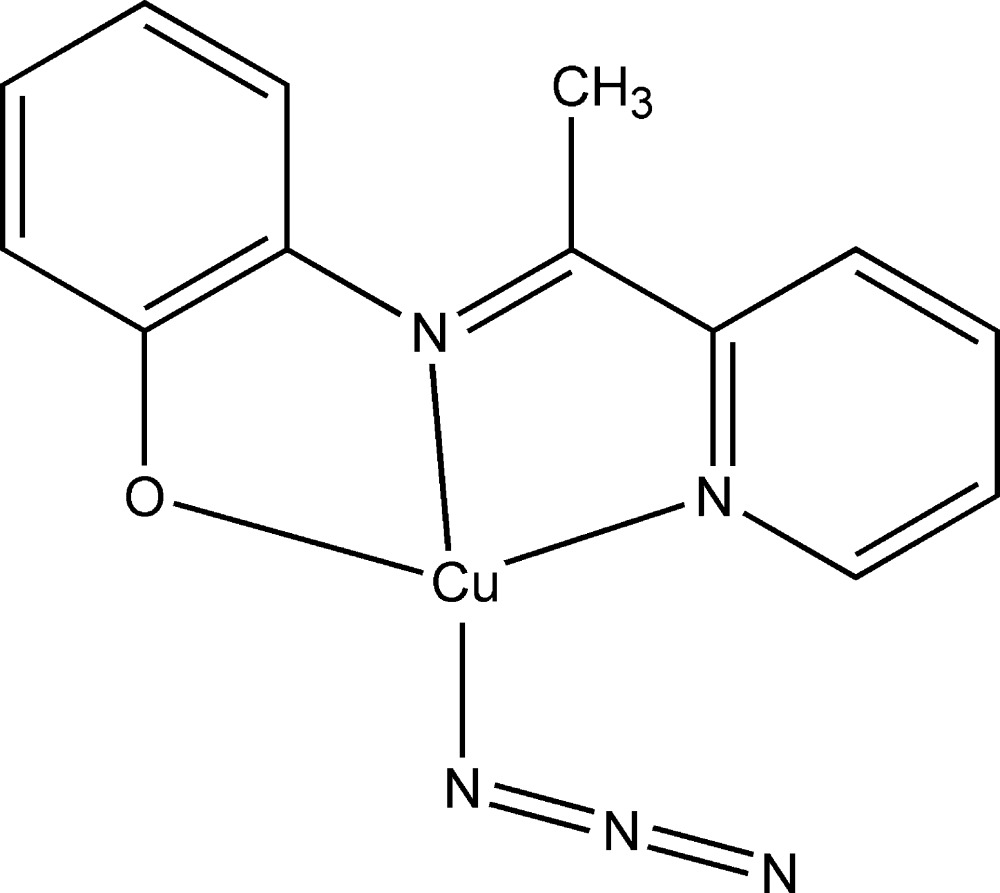


## Experimental

### 

#### Crystal data

[Cu(C_13_H_11_N_2_O)(N_3_)]
*M* *_r_* = 316.81
Monoclinic, 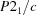

*a* = 6.5881 (3) Å
*b* = 10.1576 (3) Å
*c* = 18.3884 (7) Å
β = 92.810 (3)°
*V* = 1229.06 (8) Å^3^
*Z* = 4
Cu *K*α radiation
μ = 2.54 mm^−1^
*T* = 120 K
0.57 × 0.31 × 0.04 mm

#### Data collection

Agilent Xcalibur Gemini ultra diffractometer with Eos detector
Absorption correction: multi-scan (*CrysAlis PRO*; Agilent, 2011) ▶ *T*_min_ = 0.709, *T*_max_ = 1.000
4723 measured reflections
2357 independent reflections
2180 reflections with *I* > 2σ(*I*)
*R*_int_ = 0.021

#### Refinement

*R*[*F* ^2^ > 2σ(*F* ^2^)] = 0.032
*wR*(*F* ^2^) = 0.092
*S* = 1.05
2357 reflections
182 parameters
H-atom parameters constrained
Δρ_max_ = 0.34 e Å^−3^
Δρ_min_ = −0.40 e Å^−3^

### 

Data collection: *CrysAlis PRO* (Agilent, 2011 ▶); cell refinement: *CrysAlis PRO*; data reduction: *CrysAlis PRO*; program(s) used to solve structure: *SHELXS97* (Sheldrick, 2008 ▶); program(s) used to refine structure: *SHELXL97* (Sheldrick, 2008 ▶); molecular graphics: *ORTEP-3* (Farrugia, 2012 ▶) and *WinGX32* (Farrugia, 2012 ▶); software used to prepare material for publication: *publCIF* (Westrip, 2010 ▶).

## Supplementary Material

Crystal structure: contains datablock(s) I, global. DOI: 10.1107/S1600536813019570/lr2111sup1.cif

Structure factors: contains datablock(s) I. DOI: 10.1107/S1600536813019570/lr2111Isup2.hkl

Additional supplementary materials:  crystallographic information; 3D view; checkCIF report

## Figures and Tables

**Fig. 1.**
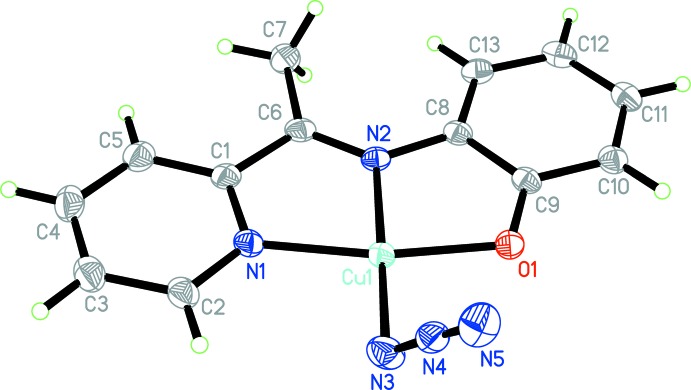
The molecular structure of the title complex, showing displacement ellipsoids at the 50% probability level.

## supplementary crystallographic information

### Comment

In the title complex, [Cu(C_13_H_11_N_2_O)(N_3_)], the Cu^II^ ion exhibits a slightly distorted square-planar coordination environment defined by the deprotonated tridentate Schiff base ligand that coordinates *via* the phenolate O, imine N and pyridyl N atoms and the N atom of azide ion (Fig. 1). The bond angles around Cu^II^ ion are slightly distorted from those of regular square-planar and range from 81.53 (7) to 175.89 (7)°. The [CuN_3_O] square plane and the aryl and pyridyl rings in the Schiff base are almost coplanar. The dihedral angles among these three planes are 3.55 (6)°, 4.70 (5)° and 4.99 (7)°. The bond distances to the central copper [Cu—N_py_ = 1.9775 (16) Å, Cu—N_imine_ = 1.9682 (16) Å, Cu—N_azide_ = 1.9470 (18) Å, Cu—O_phenolic_ = 1.9133 (14) Å] (Table 1) are similar to those in complexes [Cu(C_14_H_13_N_2_O_2_)(N_3_)] and [Cu(C_13_H_10_ClN_2_O)Cl] (Sun, 2008; Wang *et al.*, 2012). The bond distances and bond angles in azide ion bound to Cu^II^ ion are similar to those in complexes [Cu(C_14_H_13_N_2_O_2_)(N_3_)] and [Cu(C_16_H_23_N_2_O)(N_3_)] (Sun, 2008; Talukder *et al.*, 2004). The N3—N4 bond [1.201 (3) Å] in the complex is longer than the N4—N5 bond [1.157 (3) Å]. The NNN moiety is nearly linear and shows a bent coordination mode with the Cu^II^ ion [(N3—N4—N5/Cu1—N3—N4 = 177.4 (2)/117.06 (15)°].

### Experimental

The tridentate Schiff base ligand was prepared according to literature procedure (Shita *et al.*, 2009). To a hot methanolic solution (20 ml) of Cu(CCl_3_COO)_2_.6H_2_O (0.484 g, 1.0 mmol), the ligand (1.0 mmol) was added, which produced immediately an intensely green solution. The solution was then heated to boiling and then, an aqueous solution (10 ml) of sodium azide (0.065 g, 1 mmol) was added dropwise slowly over 15 min in hot condition. After the completion of addition of sodium azide, the resulting solution was kept under boiling for another 10 min. On cooling and after slow evaporation of the solution, the dark-green plate-shaped single crystals of the complex were separated out in 3 d. The crystals were filtered off and washed with water and dried in air.

### Refinement

H atoms were placed at calculated positions (C—H = 0.95–0.98 Å) and were included in the refinement in the riding-model approximation, with *U*iso(H) = 1.2*U*eq(C) and 1.5 *U*eq(C)

### Crystal data

**Table:**

[Cu(C_13_H_11_CuN_2_O)(N_3_)]	*F*(000) = 644
*M**_r_* = 316.81	*D*_x_ = 1.712 Mg m^−^^3^
Monoclinic, *P*2_1_/*c*	Cu *K*α radiation, λ = 1.54184 Å
Hall symbol: -P 2ybc	Cell parameters from 2697 reflections
*a* = 6.5881 (3) Å	θ = 4.4–71.9°
*b* = 10.1576 (3) Å	µ = 2.54 mm^−^^1^
*c* = 18.3884 (7) Å	*T* = 120 K
β = 92.810 (3)°	Plate, green
*V* = 1229.06 (8) Å^3^	0.57 × 0.31 × 0.04 mm
*Z* = 4	

### Data collection

**Table:**

Agilent Xcalibur Gemini ultra diffractometer with Eos detector	2357 independent reflections
Radiation source: Enhance Ultra (Cu) X-ray Source	2180 reflections with *I* > 2σ(*I*)
Mirror monochromator	*R*_int_ = 0.021
Detector resolution: 16.1183 pixels mm^-1^	θ_max_ = 72.0°, θ_min_ = 4.8°
ω scans	*h* = −8→6
Absorption correction: multi-scan (*CrysAlis PRO*; Agilent, 2011)	*k* = −11→12
*T*_min_ = 0.709, *T*_max_ = 1.000	*l* = −21→22
4723 measured reflections	

### Refinement

**Table:**

Refinement on *F*^2^	Primary atom site location: structure-invariant direct methods
Least-squares matrix: full	Secondary atom site location: difference Fourier map
*R*[*F*^2^ > 2σ(*F*^2^)] = 0.032	Hydrogen site location: inferred from neighbouring sites
*wR*(*F*^2^) = 0.092	H-atom parameters constrained
*S* = 1.05	*w* = 1/[σ^2^(*F*_o_^2^) + (0.0589*P*)^2^ + 0.5226*P*] where *P* = (*F*_o_^2^ + 2*F*_c_^2^)/3
2357 reflections	(Δ/σ)_max_ < 0.001
182 parameters	Δρ_max_ = 0.34 e Å^−^^3^
0 restraints	Δρ_min_ = −0.40 e Å^−^^3^

### Special details

**Table:**

Experimental. CrysAlisPro, Agilent Technologies, Version 1.171.35.21 (release 20-01-2012 CrysAlis171 .NET) (compiled Jan 23 2012,18:06:46). Empirical absorption correction using spherical harmonics, implemented in SCALE3 ABSPACK scaling algorithm.
Geometry. All e.s.d.'s (except the e.s.d. in the dihedral angle between two l.s. planes) are estimated using the full covariance matrix. The cell e.s.d.'s are taken into account individually in the estimation of e.s.d.'s in distances, angles and torsion angles; correlations between e.s.d.'s in cell parameters are only used when they are defined by crystal symmetry. An approximate (isotropic) treatment of cell e.s.d.'s is used for estimating e.s.d.'s involving l.s. planes.
Refinement. Refinement of *F*^2^ against ALL reflections. The weighted *R*-factor *wR* and goodness of fit *S* are based on *F*^2^, conventional *R*-factors *R* are based on *F*, with *F* set to zero for negative *F*^2^. The threshold expression of *F*^2^ > σ(*F*^2^) is used only for calculating *R*-factors(gt) *etc*. and is not relevant to the choice of reflections for refinement. *R*-factors based on *F*^2^ are statistically about twice as large as those based on *F*, and *R*-factors based on ALL data will be even larger.

### Fractional atomic coordinates and isotropic or equivalent isotropic displacement parameters (Å^2^)

**Table:**

	*x*	*y*	*z*	*U*_iso_*/*U*_eq_	
C11	0.1572 (3)	0.7721 (2)	0.10880 (11)	0.0224 (4)	
H11	0.1311	0.8364	0.1447	0.027*	
C12	0.1511 (3)	0.80806 (19)	0.03531 (13)	0.0238 (4)	
H12	0.1217	0.8965	0.0216	0.029*	
C13	0.1878 (3)	0.7152 (2)	−0.01764 (11)	0.0214 (4)	
H13	0.1842	0.7401	−0.0675	0.026*	
N3	0.3338 (3)	0.14937 (18)	0.06852 (10)	0.0267 (4)	
N4	0.1971 (3)	0.12200 (16)	0.10667 (9)	0.0225 (4)	
N5	0.0684 (3)	0.09080 (19)	0.14363 (10)	0.0300 (4)	
C1	0.3018 (3)	0.3279 (2)	−0.13806 (11)	0.0190 (4)	
C2	0.3721 (3)	0.1131 (2)	−0.10036 (11)	0.0233 (4)	
H2	0.3975	0.0518	−0.0620	0.028*	
C3	0.3734 (3)	0.0698 (2)	−0.17231 (12)	0.0270 (4)	
H3	0.3992	−0.0201	−0.1829	0.032*	
C4	0.3368 (3)	0.1587 (2)	−0.22775 (12)	0.0281 (5)	
H4	0.3346	0.1309	−0.2771	0.034*	
C5	0.3030 (3)	0.2905 (2)	−0.21045 (11)	0.0250 (4)	
H5	0.2809	0.3539	−0.2480	0.030*	
C6	0.2590 (3)	0.4639 (2)	−0.11276 (10)	0.0190 (4)	
C7	0.2071 (3)	0.5713 (2)	−0.16602 (11)	0.0269 (4)	
H7A	0.0753	0.6095	−0.1553	0.040*	
H7B	0.2001	0.5351	−0.2155	0.040*	
H7C	0.3120	0.6397	−0.1622	0.040*	
C8	0.2306 (3)	0.58395 (19)	0.00240 (10)	0.0176 (4)	
C9	0.2387 (3)	0.54692 (19)	0.07755 (10)	0.0181 (4)	
C10	0.2008 (3)	0.6441 (2)	0.12986 (11)	0.0208 (4)	
H10	0.2053	0.6214	0.1800	0.025*	
N1	0.3362 (2)	0.23872 (17)	−0.08411 (9)	0.0193 (3)	
N2	0.2667 (2)	0.47613 (16)	−0.04273 (9)	0.0172 (3)	
O1	0.2789 (2)	0.42425 (13)	0.09719 (7)	0.0201 (3)	
Cu1	0.31041 (4)	0.31370 (3)	0.014256 (14)	0.01721 (13)	

### Atomic displacement parameters (Å^2^)

**Table:**

	*U*^11^	*U*^22^	*U*^33^	*U*^12^	*U*^13^	*U*^23^
C11	0.0158 (9)	0.0196 (10)	0.0318 (11)	−0.0014 (7)	0.0007 (8)	−0.0067 (8)
C12	0.0179 (10)	0.0169 (10)	0.0362 (12)	−0.0003 (7)	−0.0015 (8)	−0.0001 (8)
C13	0.0173 (10)	0.0203 (10)	0.0262 (10)	−0.0010 (7)	−0.0023 (8)	0.0030 (8)
N3	0.0297 (10)	0.0213 (8)	0.0294 (9)	0.0053 (7)	0.0047 (8)	0.0048 (7)
N4	0.0305 (9)	0.0141 (8)	0.0221 (8)	0.0025 (7)	−0.0051 (7)	−0.0003 (6)
N5	0.0361 (10)	0.0259 (9)	0.0278 (9)	−0.0051 (8)	0.0021 (8)	0.0035 (8)
C1	0.0107 (9)	0.0248 (10)	0.0213 (9)	0.0000 (7)	0.0001 (7)	0.0001 (8)
C2	0.0186 (9)	0.0242 (10)	0.0271 (10)	0.0020 (8)	0.0013 (7)	−0.0035 (8)
C3	0.0209 (10)	0.0283 (11)	0.0320 (11)	−0.0001 (8)	0.0019 (8)	−0.0091 (9)
C4	0.0211 (10)	0.0381 (12)	0.0253 (10)	−0.0010 (9)	0.0020 (8)	−0.0096 (9)
C5	0.0190 (10)	0.0330 (11)	0.0230 (10)	0.0007 (8)	0.0010 (8)	−0.0004 (9)
C6	0.0117 (8)	0.0239 (10)	0.0215 (9)	0.0010 (7)	0.0015 (7)	0.0035 (8)
C7	0.0322 (11)	0.0283 (11)	0.0202 (9)	0.0067 (9)	0.0017 (8)	0.0039 (8)
C8	0.0117 (8)	0.0191 (9)	0.0220 (9)	−0.0007 (7)	0.0006 (7)	−0.0001 (7)
C9	0.0124 (8)	0.0184 (9)	0.0236 (9)	−0.0006 (7)	0.0017 (7)	−0.0008 (8)
C10	0.0157 (9)	0.0233 (10)	0.0233 (9)	−0.0006 (7)	0.0012 (7)	−0.0021 (8)
N1	0.0139 (7)	0.0221 (8)	0.0218 (8)	0.0013 (6)	0.0016 (6)	−0.0016 (7)
N2	0.0123 (7)	0.0193 (8)	0.0199 (7)	−0.0003 (6)	0.0000 (6)	0.0018 (6)
O1	0.0241 (7)	0.0180 (7)	0.0182 (6)	0.0025 (5)	0.0011 (5)	0.0012 (5)
Cu1	0.0182 (2)	0.01587 (19)	0.01753 (19)	0.00191 (10)	0.00069 (12)	0.00060 (10)

### Geometric parameters (Å, º)

**Table:**

C11—C10	1.383 (3)	C3—H3	0.9500
C11—C12	1.399 (3)	C4—C5	1.396 (3)
C11—H11	0.9500	C4—H4	0.9500
C12—C13	1.385 (3)	C5—H5	0.9500
C12—H12	0.9500	C6—N2	1.292 (3)
C13—C8	1.408 (3)	C6—C7	1.495 (3)
C13—H13	0.9500	C7—H7A	0.9800
N3—N4	1.201 (3)	C7—H7B	0.9800
N3—Cu1	1.9470 (18)	C7—H7C	0.9800
N4—N5	1.157 (3)	C8—N2	1.402 (3)
C1—N1	1.354 (3)	C8—C9	1.431 (3)
C1—C5	1.385 (3)	C9—O1	1.320 (2)
C1—C6	1.489 (3)	C9—C10	1.409 (3)
C2—N1	1.334 (3)	C10—H10	0.9500
C2—C3	1.395 (3)	N1—Cu1	1.9775 (16)
C2—H2	0.9500	N2—Cu1	1.9682 (16)
C3—C4	1.375 (3)	O1—Cu1	1.9134 (14)
			
C10—C11—C12	120.79 (18)	C6—C7—H7A	109.5
C10—C11—H11	119.6	C6—C7—H7B	109.5
C12—C11—H11	119.6	H7A—C7—H7B	109.5
C13—C12—C11	120.23 (19)	C6—C7—H7C	109.5
C13—C12—H12	119.9	H7A—C7—H7C	109.5
C11—C12—H12	119.9	H7B—C7—H7C	109.5
C12—C13—C8	120.02 (19)	N2—C8—C13	128.50 (18)
C12—C13—H13	120.0	N2—C8—C9	111.54 (17)
C8—C13—H13	120.0	C13—C8—C9	119.96 (18)
N4—N3—Cu1	117.06 (14)	O1—C9—C10	120.94 (18)
N5—N4—N3	177.4 (2)	O1—C9—C8	120.65 (17)
N1—C1—C5	120.84 (19)	C10—C9—C8	118.41 (18)
N1—C1—C6	114.76 (17)	C11—C10—C9	120.59 (19)
C5—C1—C6	124.37 (19)	C11—C10—H10	119.7
N1—C2—C3	121.6 (2)	C9—C10—H10	119.7
N1—C2—H2	119.2	C2—N1—C1	120.03 (17)
C3—C2—H2	119.2	C2—N1—Cu1	126.68 (14)
C4—C3—C2	119.1 (2)	C1—N1—Cu1	113.19 (14)
C4—C3—H3	120.4	C6—N2—C8	131.76 (18)
C2—C3—H3	120.4	C6—N2—Cu1	116.57 (14)
C3—C4—C5	119.0 (2)	C8—N2—Cu1	111.37 (12)
C3—C4—H4	120.5	C9—O1—Cu1	111.35 (12)
C5—C4—H4	120.5	O1—Cu1—N3	95.95 (7)
C1—C5—C4	119.3 (2)	O1—Cu1—N2	85.04 (6)
C1—C5—H5	120.3	N3—Cu1—N2	175.89 (7)
C4—C5—H5	120.3	O1—Cu1—N1	166.56 (7)
N2—C6—C1	113.71 (17)	N3—Cu1—N1	97.49 (8)
N2—C6—C7	125.38 (19)	N2—Cu1—N1	81.53 (7)
C1—C6—C7	120.89 (17)		
			
C10—C11—C12—C13	0.3 (3)	C7—C6—N2—C8	0.6 (3)
C11—C12—C13—C8	0.3 (3)	C1—C6—N2—Cu1	−4.7 (2)
Cu1—N3—N4—N5	166 (5)	C7—C6—N2—Cu1	173.67 (15)
N1—C2—C3—C4	0.0 (3)	C13—C8—N2—C6	−4.9 (3)
C2—C3—C4—C5	1.1 (3)	C9—C8—N2—C6	174.29 (18)
N1—C1—C5—C4	1.1 (3)	C13—C8—N2—Cu1	−178.26 (16)
C6—C1—C5—C4	−177.32 (19)	C9—C8—N2—Cu1	0.96 (18)
C3—C4—C5—C1	−1.6 (3)	C10—C9—O1—Cu1	177.20 (14)
N1—C1—C6—N2	1.7 (2)	C8—C9—O1—Cu1	−2.2 (2)
C5—C1—C6—N2	−179.81 (18)	C9—O1—Cu1—N3	−173.93 (13)
N1—C1—C6—C7	−176.72 (17)	C9—O1—Cu1—N2	2.06 (12)
C5—C1—C6—C7	1.7 (3)	C9—O1—Cu1—N1	5.2 (3)
C12—C13—C8—N2	178.35 (18)	N4—N3—Cu1—O1	56.36 (17)
C12—C13—C8—C9	−0.8 (3)	N4—N3—Cu1—N2	−47.4 (11)
N2—C8—C9—O1	0.8 (2)	N4—N3—Cu1—N1	−123.42 (16)
C13—C8—C9—O1	−179.90 (17)	C6—N2—Cu1—O1	−176.11 (14)
N2—C8—C9—C10	−178.59 (16)	C8—N2—Cu1—O1	−1.67 (12)
C13—C8—C9—C10	0.7 (3)	C6—N2—Cu1—N3	−72.0 (10)
C12—C11—C10—C9	−0.4 (3)	C8—N2—Cu1—N3	102.5 (10)
O1—C9—C10—C11	−179.47 (17)	C6—N2—Cu1—N1	4.61 (14)
C8—C9—C10—C11	−0.1 (3)	C8—N2—Cu1—N1	179.05 (13)
C3—C2—N1—C1	−0.6 (3)	C2—N1—Cu1—O1	177.1 (2)
C3—C2—N1—Cu1	175.50 (15)	C1—N1—Cu1—O1	−6.5 (4)
C5—C1—N1—C2	0.1 (3)	C2—N1—Cu1—N3	−3.81 (17)
C6—C1—N1—C2	178.62 (16)	C1—N1—Cu1—N3	172.56 (14)
C5—C1—N1—Cu1	−176.55 (15)	C2—N1—Cu1—N2	−179.78 (17)
C6—C1—N1—Cu1	2.0 (2)	C1—N1—Cu1—N2	−3.41 (13)
C1—C6—N2—C8	−177.73 (17)		
